# Capsaicin exerts synergistic antitumor effect with sorafenib in hepatocellular carcinoma cells through AMPK activation

**DOI:** 10.18632/oncotarget.21196

**Published:** 2017-09-23

**Authors:** Alicia Bort, Elena Spínola, Nieves Rodríguez-Henche, Inés Díaz-Laviada

**Affiliations:** ^1^ Department of Systems Biology, School of Medicine and Heath Sciences, University of Alcala, Alcalá de Henares, E-28871, Madrid, Spain; ^2^ Chemical Research Institute Andrés M. del Río (IQAR), University of Alcala, Alcalá de Henares, E-28871, Madrid, Spain

**Keywords:** capsaicin, sorafenib, AMPK, Akt, hepatocellular carcinoma

## Abstract

In this study, we investigated the antitumoral effects of combined treatment using sorafenib and capsaicin in hepatocellular carcinoma (HCC) cells. Here we showed that the combination of the two drugs had a much stronger inhibitory effect on both HepG2 and Huh-7 human HCC cells growth than either drug alone. The isobolograms demonstrated that the combinations investigated in this study produced a synergistic interaction. In the combination treatment using capsaicin and sorafenib, increased apoptosis, followed by the activation of caspase-9 and PARP, was observed. In addition, the present study demonstrated that sorafenib treatment induces activation of Akt, probably as a mechanism of resistance, whereas capsaicin inhibits Akt providing a possible pathway whereby capsaicin sensitizes to sorafenib in HCC cells. Moreover, capsaicin singly and the combination of capsaicin and sorafenib induce AMPK activation and Acetyl CoA carboxylase phosphorylation in HCC cells. Knocking down of AMPK by selective siRNA abrogates capsaicin-induced Akt inhibition, suggesting the involvement of AMPK in the antiproliferative effect. *In vivo* experiments further showed that that the anti-tumor effect of sorafenib was enhanced by its combination with 2.5 mg/Kg of capsaicin. Overall, these results show that combined treatment with capsaicin and sorafenib might improve sorafenib sensitivity and therefore it represents a promising and attractive strategy for the treatment of HCC.

## INTRODUCTION

Hepatocellular carcinoma (HCC) is a highly aggressive solid malignancy and the third cause of cancer related deaths [1]. Its incidence is rising globally at an alarming rate [2], making HCC the fifth most common cancer in men and the seventh most common cancer in women [1, 3]. In spite of well-established monitoring programs in patients with risk, most tumors are diagnosed at intermediate-advanced stage, and only palliative measured can be applied. Current treatments applicable at early stages of tumor development include tumor resection, liver transplantation and chemoembolization. The only approved systemic treatment for advanced HCC is sorafenib, a multi-kinase inhibitor that targets c-Raf, B-Raf and VEGF receptor, among others. However, despite the overall survival increase and the better outcome that have been obtained with sorafenib treatment [4], many patients have to adopt dose reduction or terminate the use of sorafenib because of adverse effects such as hand-foot syndrome [5], bleeding of gastrointestinal tract [4, 6] or effects on liver function. In addition acquired resistance develops more often than desired [7].

There are many proposed mechanisms underlying sorafenib resistance including elevated expression of drug efflux transporters, sustained activation of phosphatidylinositol 3-kinase (PI3K)/Akt signaling pathway, upregulation of cell survival and proliferation genes and downregulation of tumor suppression molecules and cell cycle arrest genes [7]. Moreover, the proliferation and differentiation of liver cancer stem cells seems to be a new mechanism for sorafenib resistance in HCC [7]. Thus, targeting pathways involved in sorafenib resistance would sensitize tumors to sorafenib -induced antiproliferation. Therefore, new strategies that enhance the antitumor effect of sorafenib are urgently required. In the recent years, novel drugs have been tried both in first-line and second-line therapy for advanced HCC. Until now, none of these have proven to be better than sorafenib in first-line trials, in terms of survival [8].

There is a growing body of evidence that phytochemicals possess anticancer properties in HCC [9]. Among them, the pungent ingredient of hot peppers of genus *Capsicum*, Capsaicin (N-vanillyl-8-methyl-1-nonenamide), has revealed as a promising chemotherapeutic agent. Capsaicin exhibits, anti-proliferative and anti-angiogenic actions inhibiting the development and progression of many types of tumors through multiple mechanisms. Capsaicin can induce cell-cycle arrest or apoptosis or may inhibit proliferation in a variety of cancer cells through generation of ROS and persistent disruption of mitochondrial membrane potential [10, 11]. In HCC cells, capsaicin inhibits proliferation through the induction of autophagy and apoptosis [12]. Capsaicin induced apoptosis in HCC cells involves increase of intracellular calcium concentration, expression of heme oxygenase-1 [13], production of reactive oxygen species (ROS) and activation of caspase-3 [14, 15].

In this study we explored the *in vivo* and *in vitro* antitumor effects of capsaicin in combination with sorafenib and the underlying signaling pathways. We showed that the combined treatment of HCC cells with subtoxic doses of capsaicin and Sorafenib dramatically induced HCC cell death. Moreover, *in vivo* administration of capsaicin and sorafenib in xenograft HCC tumors had synergic antiproliferative effect compared with either compound alone. Hence we conclude that a combined treatment with sorafenib and capsaicin may synergistically stimulate and accelerate the antitumor effect of sorafenib.

## RESULTS

### Capsaicin and sorafenib exert a synergistic cytotoxic effect on HCC cells

**Figure 1:**
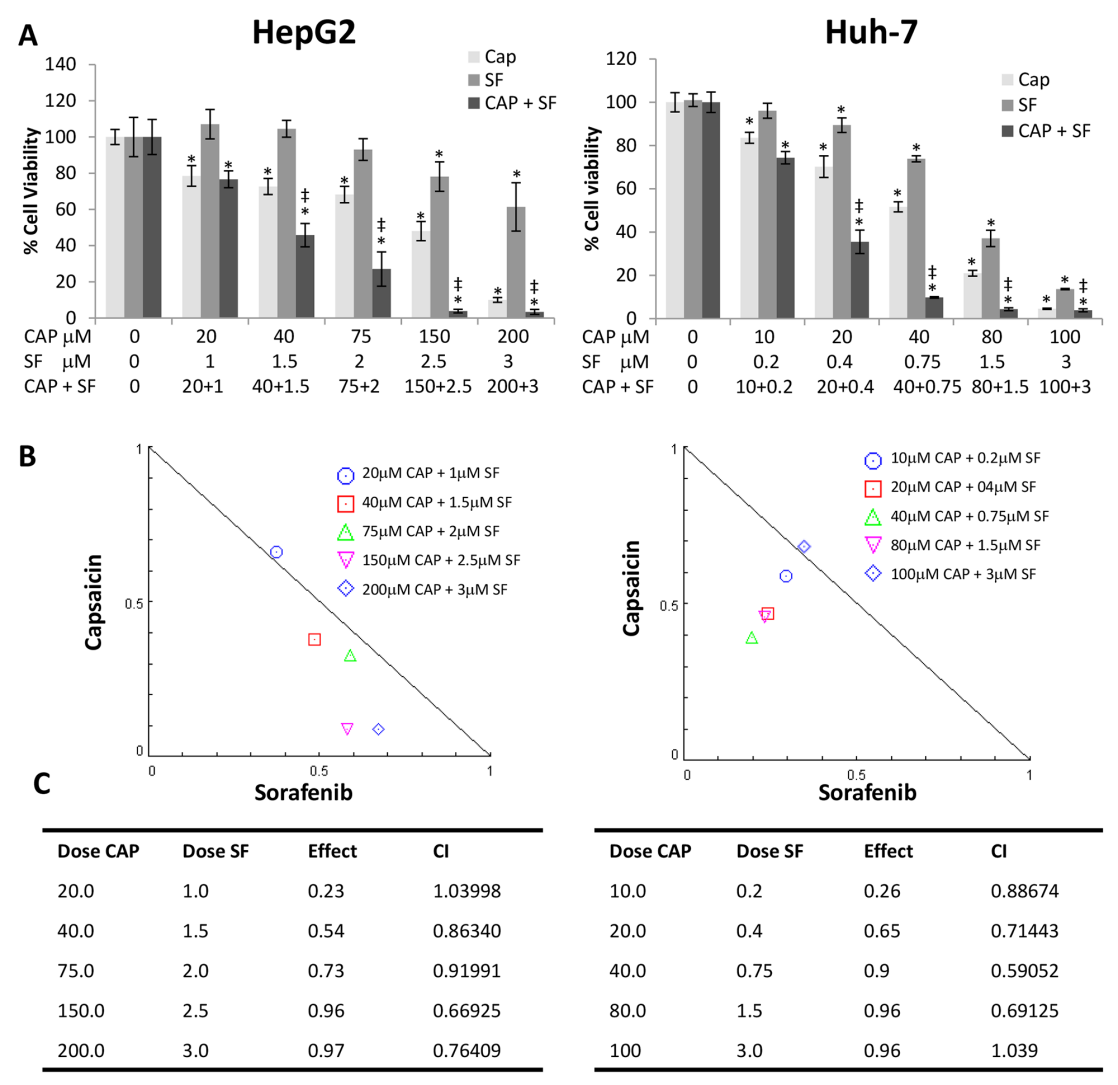
Capsaicin and sorafenib synergistically inhibited hepatocellular carcinoma (HCC) cells viability **(A)**, Effect of the combination of different doses of capsaicin and sorafenib on HCC cell viability. Cells were treated with capsaicin or sorafenib alone, or in combination at the indicated concentrations, for 24 h. Cell viabilities were determined by MTT assay and expressed as percentages of those of control (DMSO treatment). ^*^, p<0.0001 significant difference between treated and control cells by two-way ANOVA and Dunnett’s multiple comparisons test; ‡, p<0.0001 indicate significant interaction between CAP and SF treatment. Experiments were run in triplicate and carried out at least two times on separate occasions. **(B)**, Isobologram representation of the cell viability assay with the combination of both drugs. **(C)**, Combination index (CI) of drug interaction at the different concentrations assayed is shown in the table.

To examine the effect of capsaicin and sorafenib on HCC cell proliferation, MTT cell viability assays were carried out using HepG2 and Huh-7 cells. Exposure to different concentrations of capsaicin or sorafenib caused a significant concentration-dependent decrease in the cell viability compared with the negative control group (Figure 1A). Analysis of dose-response data allowed calculation of inhibitory half doses (IC50) for viability. Comparison of the IC50s results revealed that capsaicin and sorafenib had a stronger effect on Huh-7 than HepG2 cells since capsaicin inhibited cell viability with an IC50 of 150μM in HepG2 and 40μM in Huh-7 cells and sorafenib with an IC50 of 2.5μM in HepG2 and 0.75μM in Huh-7 cells. The combination capsaicin with sorafenib produced a higher decrease in the cell viability as compared to either monotherapy treatment with either drug in both cell lines (Figure 1A).

To determine whether both drugs had a synergistic effect on cell proliferation, we used the Combination-index (CI) method which is a mathematical and quantitative representation of a two-drug pharmacologic interaction [16, 17]. Using data from the growth inhibitory experiments and Compusyn© software [16], CI values were generated. CI allows the quantification of synergism or antagonism for two drugs where CI of 1 indicates an additive effect, whereas a CI < 1 or CI > 1 indicates synergism or antagonism, respectively. Combination-index showed a potent synergy of cell killing in both cell lines, at four out of the five combinations used. Likewise, isobologram for the combination of capsaicin and sorafenib in HepG2 and Huh-7 cell lines shows that four of the combination data points fall on the lower left, indicating synergism (Figure 1B and 1C).

**Figure 2:**
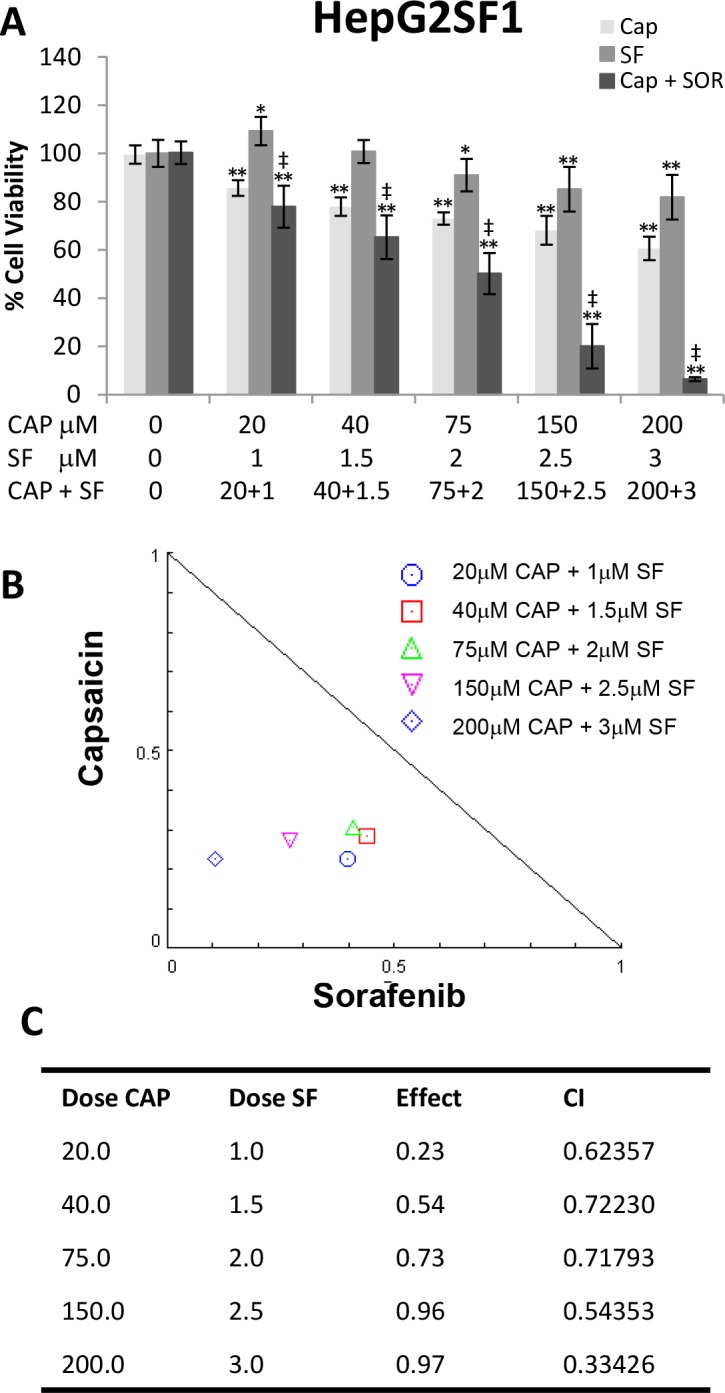
Capsaicin sensitizes sorafenib-resistant HepG2SF1 cells to sorafenib **(A)**, Effect of the combination of different doses of capsaicin and sorafenib on HepG2SF1 cell viability. Cells were treated with capsaicin or sorafenib alone, or in combination at the indicated concentrations, for 24 h. Cell viabilities were determined by MTT assay and expressed as percentages of those of control (DMSO treatment). ^*^, p<0.01 and ^**^, p<0.0001 significant difference between treated and control cells by two-way ANOVA and Dunnett’s multiple comparisons test; ‡, p<0.0001 indicate significant interaction between CAP and SF treatment. Experiments were run in triplicate and carried out at least two times on separate occasions. **(B)**, Isobologram representation of the cell viability assay with the combination of both drugs. **(C)**, Combination index (CI) of drug interaction at the different concentrations assayed is shown in the table.

To further investigate whether capsaicin could sensitize hepatocellular cells to sorafenib, we generated the sorafenib-resistant cell line HepG2SF1 by y adapting the HepG2 cells to grow in the presence of sorafenib. Sorafenib-resistant cells were incubated with increasing concentrations of CAP, SF or their combination. As expected, sorafenib treatment was not as effective as in HepG2 cells in reducing cell viability (Figure 2). Nevertheless, capsaicin inhibited cell viability as in HepG2 cells and interestingly capsaicin re-sensitized sorafenib-resistant cells to sorafenib treatment, as indicated by decreased cell viability (Figure 2). Moreover, CompuSyn© analysis revealed that the CI was less than 1 in the five combinations tested, indicating synergism (Figure 2).

### The combination of capsaicin and sorafenib induced apoptosis in HCC cells

**Figure 3:**
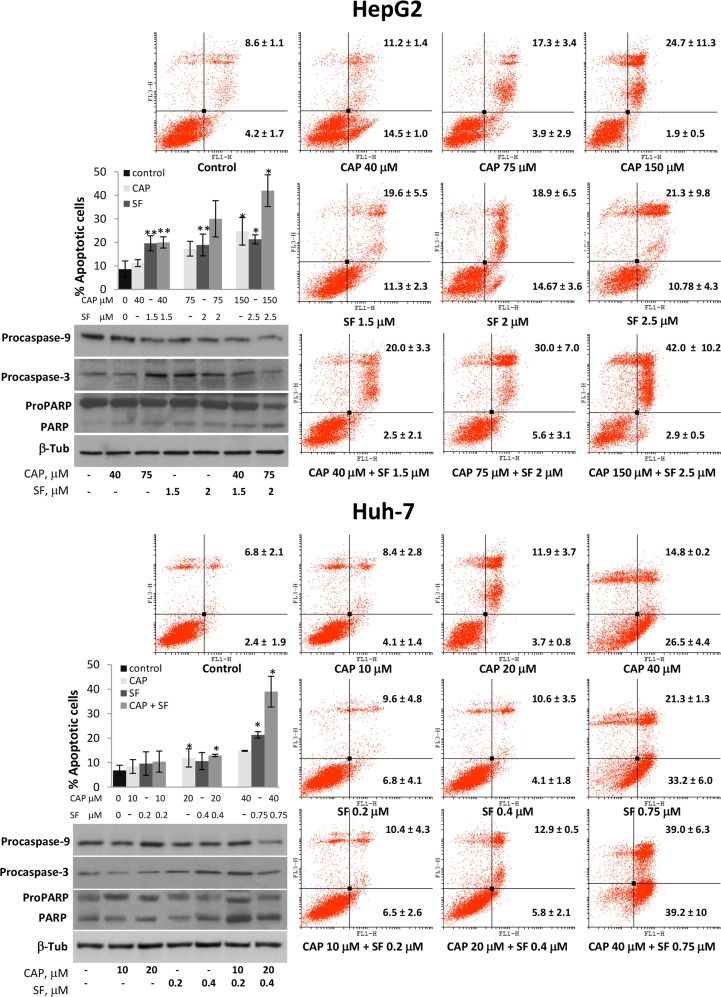
Combined treatment of capsaicin and sorafenib increased HCC cells apoptosis HepG2 and Huh-7 cells were treated with vehicle (control) or the indicated doses of capsaicin, sorafenib or a combination for 24h and then stained with Annexin V and PI. The graphs represent PI fluorescence (Y axe) versus Annexin V fluorescence (x axe). The early apoptotic cells (AnnexinV-positive, PI-negative cells) and the late apoptotic cells (Annexin V-positive, PI-positive cells) are indicated as the percentage of gated cells. Histogram represents the late apoptotic cells for each dose. Data are the mean ± SEM of three experiments. ^*^p<0.05 and ^**^p<0.01 vs control compared by the Student’s t test. Below, HepG2 and HuH-7 cells were incubated with the indicated doses of capsaicin, sorafenib or both compounds for 24h and levels of procaspase-9, procaspase-3 and PARP were determined by Western blot. β-Tubulin (β-Tub) was determined as loading control. Experiments were performed three times.

We next explored the effects of capsaicin and sorafenib on tumor cell apoptosis. In order to avoid the excessive cytotoxic effect produced by the highest doses, for these experiments we choose the combination of IC50 doses and two lower combinations. Flow cytometry analysis showed that 24 hour treatment of the cells with increasing doses of capsaicin or sorafenib alone had a little effect on HepG2 and Huh-7 tumor cell apoptosis (Figure 3). In contrast, combination of capsaicin and sorafenib induced a significant increase in late apoptotic cells (Annexin-V positive, IP positive, upper right panel) (Figure 3). Cell cycle analysis revealed an increase in subG0 cell population when cells are treated with combined doses of capsaicin and sorafenib (Supplementary Figure 1), further confirming an apoptosis induction by both compounds when added together. We then investigated whether both compounds induced apoptosis through classical apoptotic type I cell death and caspase activation. Immunoblot analysis revealed that combined capsaicin and sorafenib at 75 μM and 2 μM respectively did markedly reduce the precursor form of caspase-9 and of caspase-3. Consistently, the cleavage of PARP, which is a substrate of caspase-3 and a well-known apoptotic hallmark, was activated by the highest combination of capsaicin and sorafenib in the tested cell lines (Figure 3).

### Capsaicin inhibits Akt and counteracts sorafenib-induced Akt activation in HCC cells

**Figure 4:**
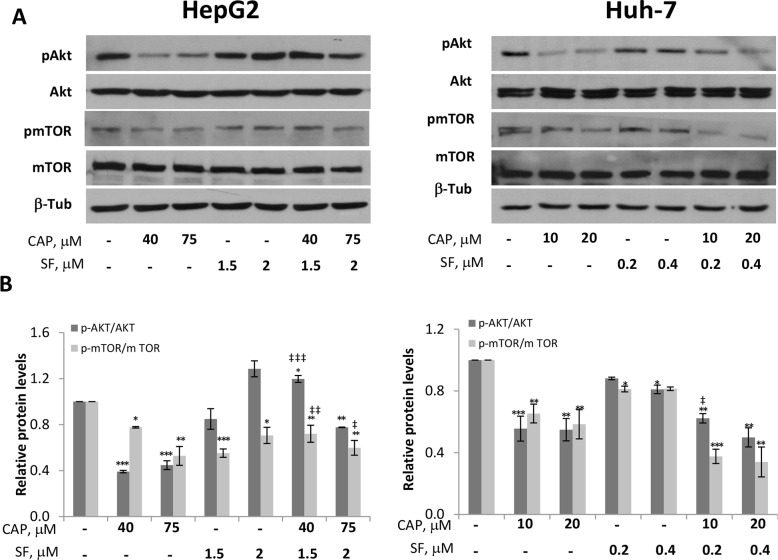
Inhibition of Akt/mTOR pathway by capsaicin in HCC cells HepG2 and Huh-7 cells were treated with capsaicin, sorafenib or both for 1 hour and levels of the phosphorylated proteins and their total forms were determined by Western blot. **(A)**, Representative Western blot on the levels of pAkt and pmTOR and their corresponding total forms. β-tubulin (β-Tub) serves as a loading control. **(B)**, The densitometric analyses of bands represented as the mean ± SD of three different experiments. ^*^, p<0.05, ^**^, p<0.01 and ^***^, p<0.001 significant difference between treated and control cells by two-way ANOVA and Dunnett’s multiple comparisons test; and ‡, p<0.05, ‡ ‡, p<0.01 and ‡ ‡ ‡, p<0.001 indicate significant interaction between CAP and SF treatment.

Many signaling pathways within the cell orchestrate proliferation and growth. Among them, the PI3K/Akt axe has revealed as key survival regulatory element frequently altered in human cancers including hepatocellular carcinoma in which contributes to the resistant phenotype [18, 19]. To define the intracellular mechanism underlying capsaicin and sorafenib synergistic inhibitory effect on cell viability, we analyzed the status of PI3K/Akt pathway. As shown in Figure 4, capsaicin treatment produced a marked inhibition of Akt phosphorylation at Ser473 at both doses tested. Accordingly, the phosphorylation of its downstream signaling protein mammalian target of rapamycin (mTOR) in ser2448 was decreased in capsaicin-treated cells. Nevertheless, sorafenib treatment did not cause such inhibition of PI3K/Akt pathway as deduced by Akt and mTOR phosphorylation (Figure 4). Interestingly, the combination of capsaicin and sorafenib decreased Akt and mTOR phosphorylation at the highest doses tested compared to sorafenib alone. This finding indicates that capsaicin counteracts sorafenib-induced PI3K/Akt activation and provides a possible explanation for the observed synergistic effect between both compounds.

### Capsaicin and combined treatment using capsaicin and sorafenib activate AMPK in HCC cells

**Figure 5:**
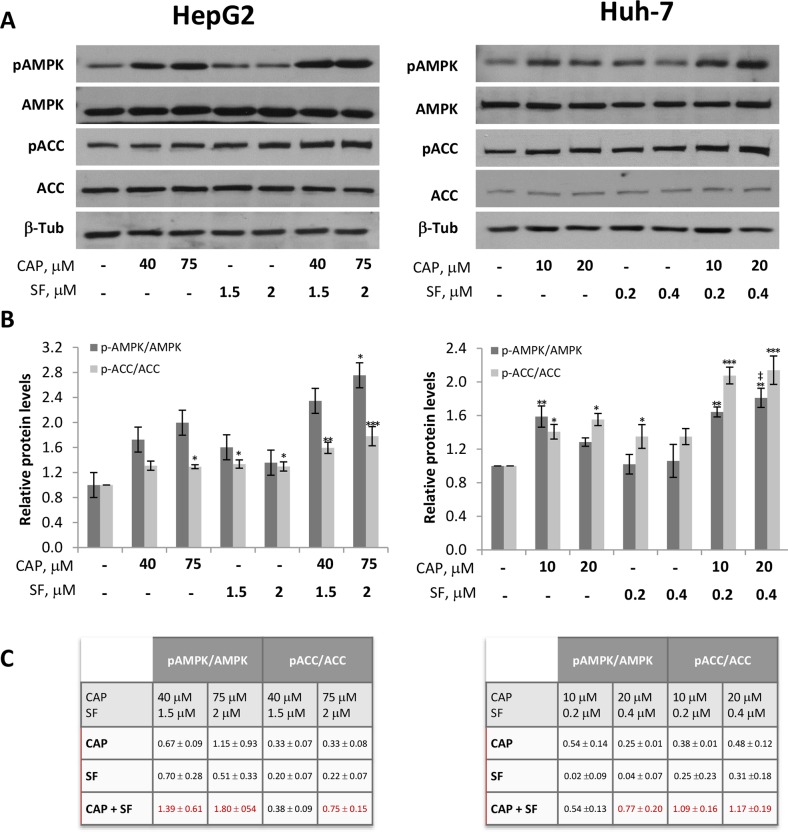
Activation of AMPK by capsaicin and sorafenib in HCC cells HepG2 and Huh-7 cells were treated with capsaicin, sorafenib or both for 1 hour and levels of the phosphorylated proteins and their total forms were determined by Western blot. **(A)** Representative Western blot on the levels of pAMPK and pACC and their corresponding total forms. β-tubulin (β-Tub) serves as a loading control. **(B)**, The densitometric analyses of bands represented as the mean ± SD of three different experiments^*^, p<0.05, ^**^, p<0.01 and ^***^, p<0.001 significant difference between treated and control cells by two-way ANOVA and Dunnett’s multiple comparisons test; and ‡, p<0.05 indicate significant interaction between CAP and SF treatment. **(C)**, Increment in protein phosphorylation when compounds were singly administered or in combination. In red when effect is synergistic.

The key metabolic sensor AMP-activated kinase (AMPK) has emerged as a relevant regulator of cell growth. AMPK phosphorylates many proteins involved in autophagy regulation as well as proteins implicated in the control of cell cycle and apoptosis, like p21 and p53, strongly suppressing cell proliferation [20]. In the last few years AMPK has been identified as a target of several nutraceutical compounds that increase AMPK phosphorylation to modulate many cell functions [21]. To investigate whether capsaicin modulates AMPK and to examine the mechanism underpinning the cytotoxicity by capsaicin, we analyzed the activation of AMPK in HCC cells. We therefore measured phosphorylation of AMPKα at its activation site Thr172 and phosphorylation of its well-known substrate Acetyl CoA Carboxylase (ACC) at Ser79, after treatment of cells with capsaicin, sorafenib, or the combination thereof. As shown in Figure 5, significant AMPK activation, as judged by AMPK phosphorylation as well as by ACC phosphorylation, was observed in the cells treated with capsaicin alone. Importantly, although sorafenib-induced AMPK activation was no significant, a marked significant AMPK activation occurred when sorafenib was combined with capsaicin in HepG2 and Huh-7 cells. Interestingly, a synergic effect could be observed in AMPK and ACC phosphorylation at the highest combination tested in both cell lines (Figure 5).

### AMPK knocking down abrogates capsaicin-induced Akt inhibition in HCC cells

**Figure 6:**
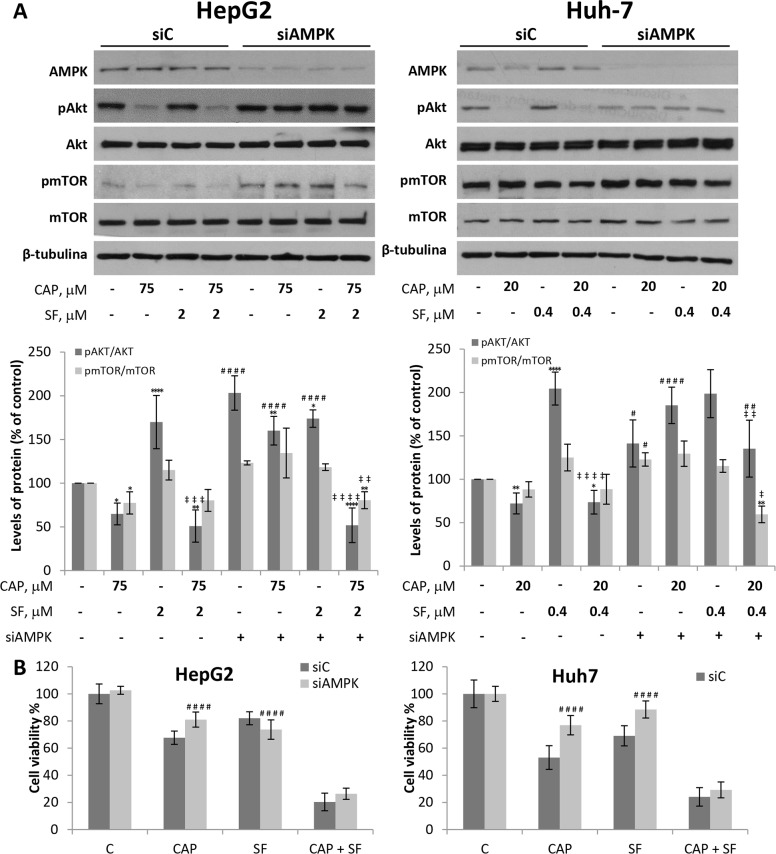
Downregulation of AMPK blocks capsaicin-induced Akt/mTOR and cell viability inhibition in HCC cells **(A)** HepG2 and Huh-7 cells were transfected with control siRNA (siC) or *ɑ*1AMPK-selective siRNA (siAMPK) and then treated with capsaicin, sorafenib or both for 1 hour. Levels of the phosphorylated proteins and their total forms were determined by Western blot. (A), Representative Western blot on the levels of AMPK, pAkt, pmTOR and their corresponding total forms is shown. β-tubulin (β-Tub) serves as a loading control.) The densitometric analyses of bands represented as the mean ± SD of four different experiments. **(B)**, Cell viability in cells transfected with control siRNA (siC) or *ɑ*1AMPK-selective siRNA (siAMPK) and then treated with capsaicin, sorafenib or both for 24 h. Cell viabilities were determined by MTT assay and expressed as percentages of those of control (DMSO treatment). ^*^, p<0.05, ^**^, p<0.01 and ^****^, p<0.0001 significant difference between treated and control cells by two-way ANOVA and Dunnett’s multiple comparisons test; ‡, p<0.05, ‡ ‡, p<0.01, ‡ ‡ ‡, p<0.001 and ‡ ‡ ‡ ‡, p<0.0001 indicate significant interaction between CAP and SF treatment; #, p<0.05, ##, p <0.01 and ####, p<0.0001 significant different between siAMPK and siC by two-way ANOVA and Sidak’s multiple comparisons test. Experiments were run in triplicate and carried out at least two times on separate occasions.

To investigate the role of AMPK in the cytotoxic effect induced by capsaicin and sorafenib, we knocked down this protein using siRNA. Downregulation of AMPK prevented capsaicin-induced Akt inhibition both in HepG2 and in Huh-7 cells (Figure 6A). This increase in Akt phosphorylation after AMPK knockdown suggests that AMPK negatively regulates Akt phosphorylation upon capsaicin treatment. In contrast, AMPK knocking down did not modify Akt activation in sorafenib-treated cells. When cells were co-treated with capsaicin and sorafenib, a significant prevention of Akt inhibition was appreciated in AMPK knocked down cells (Figure 6A).

To further understand the role of AMPK, we tested whether it is involved in the antiproliferative effect of capsaicin and sorafenib. As shown in Figure 6B, when AMPK was knocked down in HepG2 cells, capsaicin-induced cell viability decrease was significantly blocked. Likewise, in siAMPK Huh-7 transfected cells, a prevention of capsaicin and sorafenib -driven cell death was observed. AMPK knocking down in cells co-treated with capsaicin and sorafenib although not significantly, a prevention of cell death was observed. These data altogether indicate that capsaicin through AMPK activation, inhibits Akt pathway which is required for the capsaicin-induced antiproliferative effect.

### Sorafenib and capsaicin synergistically inhibit the growth of HCC tumors *in vivo*

**Figure 7:**
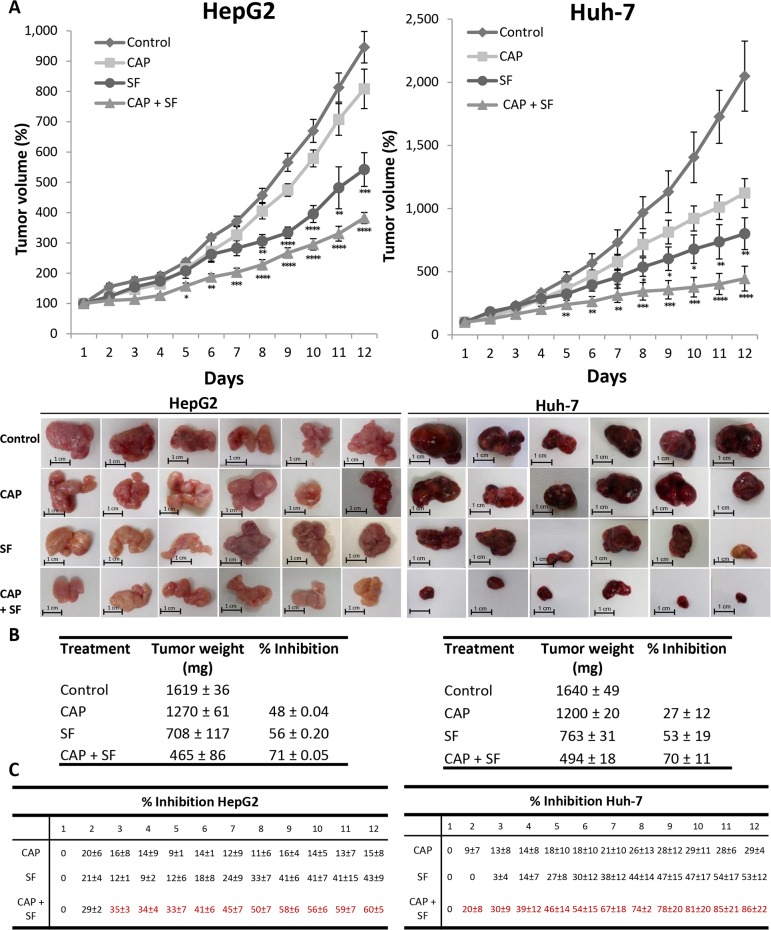
Effect of the combination of sorafenib with capsaicin in a tumor xenograft model **(A)**, The growth curves of HepG2 and Huh-7 cells as tumor xenografts in nude mice treated with vehicle (diamonds), 30 mg/Kg/day sorafenib (circles), 5 mg/Kg/day capsaicin (squares) or the combination (triangles). Error bars represent SEM; *n* = 6. I^*^, p<0.05, ^**^, p<0.01, ^***^, p<0.001 and ^****^, p<0.0001 significant difference between treated and control mice by two-way ANOVA and Tukey’s multiple comparisons test. Images of the tumors of each treatment dissected at the end of the treatment are shown below. **(B)**, Table shows tumor weights at the end of the treatment (mean ± SEM). **(C)**, Tumor growth inhibition (mean ± SEM).

**Figure 8:**
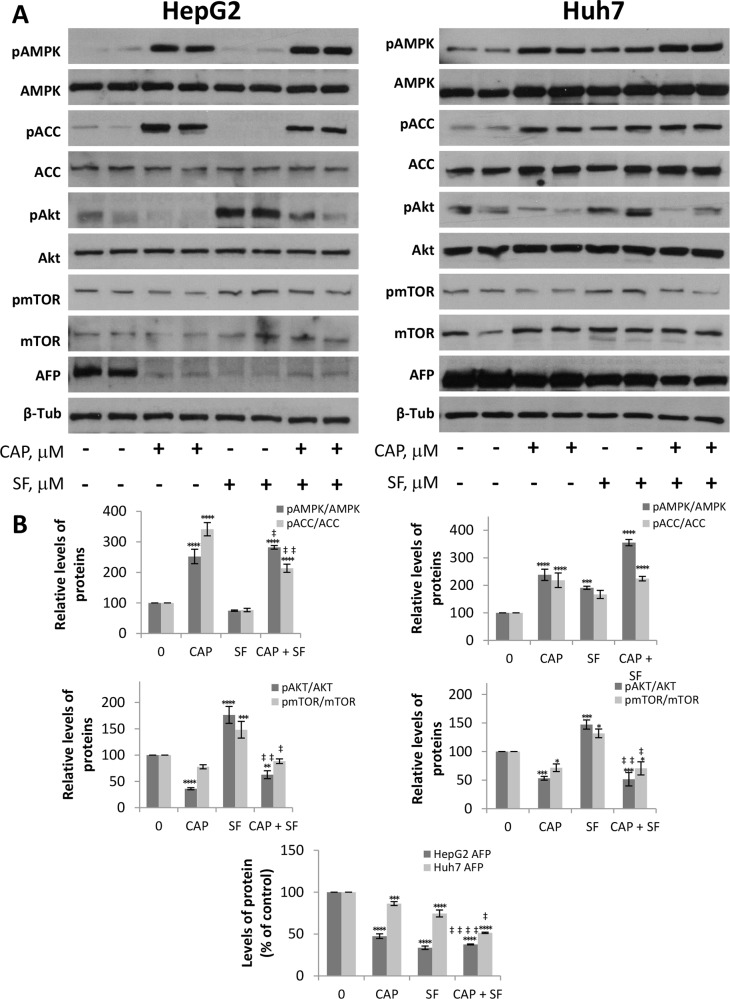
Levels of different proteins on capsaicin- and sorafenib-treated tumors Dissected tumors were homogeneized and levels of pAMPK, total AMPK, pACC, total ACC, pAkt, total Akt, pmTOR, total mTOR as well as *ɑ*-fetoprotein (AFP) were determined by Western blot. **(A)**, Image of WB of two representative tumors of each treatment. β-tubulin (β-Tub) serves as a loading control. **(B)**, The densitometric analyses of bands represented as the mean ± SD of all tumors. ^*^, p<0.05, ^**^, p<0.01, ^***^, p<0.001 and ^****^, p<0.0001 significant difference between treated and control mice by two-way ANOVA and Dunnett’s multiple comparisons test; ‡, p<0.05, ‡ ‡, p<0.01 and ‡ ‡ ‡ ‡, p<0.0001 indicate significant interaction between CAP and SF treatment.

To validate the synergism between capsaicin and sorafenib observed in HCC cells, we evaluated the antitumoral effect of the compounds alone or in combination in a mouse xenograft model of HCC generated by inoculation of HepG2 or Huh-7 cell lines. When tumors reached a volume of 70 mm^3^, animals were randomly assigned into different groups with 6 mice per cohort and treated with capsaicin at dose level of 2.5 mg/kg body weight, sorafenib at 30 mg/kg body weight, both drugs as above or the equivalent volume of vehicle. As shown in Figure 7A, daily treatment of mice with capsaicin or sorafenib alone produced a significant delay in HCC tumor progression compared to the vehicle-treated animals (control). The combination of capsaicin and sorafenib almost totally blocked tumor growth in Huh-7 cell-derived tumors and efficiently and significantly reduced tumor growth in HepG2 cell-derived tumors (Figure 7A). When compared to vehicle-treated mice, at the completion of the study, we observed that tumor weight was significantly reduced in mice treated either with capsaicin or with sorafenib as single agents (Figure 7B). However, the combination of both agents was significantly more effective than the two drugs alone leading to a 70% reduction in tumor burden. The analysis of the tumor growth inhibition by day indicates that the inhibitory effect of the co-treatment with capsaicin and sorafenib was synergistic from day 2 in HepG2 cells and from day 3 in Huh-7 cells (Figure 7C). To finally determine whether capsaicin and sorafenib activate the same signaling pathways *in vivo* than in cultured HCC cells, levels of pAMPK, pACC, pAkt, pmTOR and their corresponding total forms in the dissected tumors were analyzed by Western blot. As shown in Figure 8, capsaicin activates AMPK and inhibits Akt/mTOR pathway whereas sorafenib activates Akt/mTOR. According to the *in vitro* data, the combination of capsaicin and sorafenib activates AMPK and inhibits Akt/mTOR. Moreover, the combination of capsaicin and sorafenib decreased the expression of the HCC tumor marker alpha-fetoprotein (AFP) in the dissected tumors (Figure 8).

Altogether, these results suggest that the combination of capsaicin with sorafenib abrogates the activation of Akt induced by sorafenib alone and therefore it might represent an efficient therapeutic approach for the treatment of HCC tumors.

## DISCUSSION

Despite recent advances in the treatment of HCC, no effective systemic therapy for HCC is still available. Sorafenib is, at present, the only drug approved for the treatment of hepatocellular carcinoma but the outcome is still poor. In addition, a considerable number of patients develop resistance to sorafenib reducing their survival time. Hence, new treatments that improve sorafenib efficacy are urgently needed. In this study, we show that treatment of HCC cells with capsaicin sensitizes cells to sorafenib-induced cell death and both agents synergistically inhibit HCC cell proliferation. Apoptosis was the main mechanism for combined treatment-induced cell death, as demonstrated by Annexin-V assay and PARP activation. We showed that the combination of these two agents had synergistic effects on decreasing cell survival of HepG2 and Huh-7 cells. It is worthy to note that Huh-7 cells better responded to the combined treatment of sorafenib and capsaicin both *in vitro* and *in vivo*. Although we do not have an explanation for this difference, recent findings demonstrated that Huh-7 cells express higher levels of P-glycoprotein, OATP1B1 (organ anion transporter 1B1) and OCT1 (organ cation transporter 1) membrane transporters compared with those of HepG2 [22]. The polyspecific cation transporter OCT1 is one of the most important active influx pumps for sorafenib. Accordingly, recent research revealed that the expression of OCT1 in HCC biopsies had a positive prognostic factor for patients treated with sorafenib [23]. Hence, this suggests that sorafenib may be more efficiently transported into Huh-7 cells to target the inhibitory pathways. Whether capsaicin enters into the cell by OATP1B1 or by diffusion is a fact that needs further demonstration.

To study the mechanism underlying the synergy between capsaicin and sorafenib we selected the lower doses that had shown a synergic effect as this combinations would have a beneficial effect *in vivo* minimizing secondary non-desired effects. We show here that capsaicin treatment of HCC cells inhibited Akt and mTOR phosphorylation, and the combination of capsaicin and sorafenib counteracted the Akt and mTOR activation induced by sorafenib alone. This finding is remarkable since it is thought that activation of Akt may be responsible for mediating the acquired resistance to sorafenib in HCC cells [24, 25]. Moreover, activation of the mTOR pathway in HCC is associated with less differentiated tumors, earlier tumor recurrence, and worse survival outcome [26]. Hence, inhibition of Akt/mTOR pathway by capsaicin would increase sorafenib-induced cell death and improve its beneficial effects. Likewise, pharmacological inhibition of PI3K/mTOR showed additive antitumor effects *in vitro* and *in vivo* in Huh-7 cells [27]. In line with this, in differentiated tumor cancer biopsies a lower tumor expression of nuclear pAkt was associated with higher rate of response to sorafenib [28]. In fact, many inhibitors targeting PI3K/Akt/mTOR pathway are currently being evaluated for HCC treatment in preclinical and clinical studies [19]. The fact that inhibition of Akt by capsaicin in HCC cells abrogates sorafenib-induced Akt phosphorylation underscores the potential of a combined therapeutic approach with both agents. These results indicate that capsaicin induce downregulation of survival pathways which may cover up the resistance signals induced by sorafenib.

The present study also found that capsaicin alone induced AMPK activation and that AMPK knocking down prevents capsaicin-induced cell death. Of note, the combination treatment appeared to lead to stronger induction of AMPK signaling since a higher phosphorylation of both AMPK and ACC was found. In line with our results, it has recently demonstrated that the AMPK activator metformin could increase the sensitivity of HCC cells to sorafenib and inhibit HCC recurrence and metastasis in orthotopic mouse models [29]. Similarly, activation of AMP-activated protein kinase by retinoic acid sensitized hepatocellular carcinoma cells to apoptosis induced by sorafenib [30]. In non-small cell lung cancer the combination of sorafenib with metformin synergistically inhibited cellular proliferation [31]. All these data suggest that AMPK activation can be a strategy to sensitize cell to sorafenib-induced antiproliferative effects.

Overall, our results indicate that capsaicin treatment, by downregulation of survival pathways and upregulation of antiproliferative pathways could overcome unwanted tumor resistance and consequently help to improve the benefits of sorafenib therapy in HCC.

## MATERIALS AND METHODS

### Reagents and antibodies

Capsaicin (CAP) and Sorafenib were purchased to Sigma (St. Louis, MO, USA). Primary antibodies anti-caspase-9, anti-PARP, anti-AFP, anti-pAkt-ser473, p-mTOR-ser2448, p-AMPKα1-thr172, p-ACC-ser79 and the antibodies against the corresponding total forms were obtained from Cell Signaling Technology (Danvers, MA, USA). The anti-caspase-3 antibody was from Santa Cruz Biotechnology, Inc. (Dallas, TX, USA). (Peroxidase labeled secondary anti-mouse IgG was from Sigma (St. Louis, MO, USA) and anti-rabbit IgG was from Calbiochem (San Diego, CA, USA).

### Cell lines and cell culture

The human hepatocellular carcinoma HepG2 cells line was purchased from the American Type Culture Collection (ATCC HB-8065, Rockville, MD, USA). The human hepatoma cell line Huh-7 was kindly provided by Dr. Lisardo Boscá (instituto de Investigaciones Biomédicas Alberto Sols, Madrid). Cell lines were incubated at 37 °C in a humidified atmosphere with 5% CO2 and cultured in DMEM/10%FBS supplemented with 1% non-essential amino acids and 100 IU/mL penicillin G sodium, 100 μg/mL streptomycin sulfate, 0.25 μg/mL amphotericin B (Invitrogen, Paisley, UK).

To generate sorafenib-resistant cells, HepG2 cells were cultured continuously for 5 months with a step-wise increase of sorafenib concentrations (0.75 - 8 μM). HepG2 parental cells were cultured in parallel without sorafenib and served as control.

### Cell viability assay

A total of 5000 cells/well were seeded into 12-well plate in a final volume of 1mL. At 24 h following seeding, the medium was aspirated and replaced with fresh medium containing various concentrations of capsaicin or sorafenib or both and incubated for the indicated times at 37°C in an atmosphere containing 5% CO_2_. Vehicle control cultures received DMSO alone. The number of viable cells at the end of the incubation period was measured using a MTT (3-(4,5-dimethylthiazol-2-yl)-2,5-diphenyltetrazolium bromide) Cell Proliferation assay (Sigma, St. Louis, MO, USA). Absorbance at 490 nm was read using a microplate reader (ELX 800 Bio-Tek Intruments, INC) and subtracted with non-specific absorbance measured at 650 nm. Cell viability was calculated as a percentage compared to the control cells, which were arbitrarily assigned 100% viability. The half maximal inhibitory concentration (IC_50_) values, defined as the concentration that inhibited 50% cell growth relative to control cells, were graphically obtained from the dose-response curves.

### Flow cytometry for cell cycle and apoptosis

Flow cytometry was used to detect the distribution of cell cycle and apoptotic cells. After being cultivated with medium alone or medium containing the indicated stimuli, 10^5^ cells in 35 mm culture dish were harvested in 0.35% trypsin, collected and fixed with 70% cold ethanol at 4°C for 1h. Then, cells were centrifuged at 1500g for 5 min and incubated in 0.5 ml PBS containing 0.1 mg/ml RNase for 30 min at 37°C. DNA staining was performed adding 5 μl propidium iodide (Invitrogen, Eugene, Oregon, OR, USA). Apoptosis was evaluated at 24 h following treatment using an Annexin V-fluorescein isothiocyanate (FITC) Apoptosis Detection kit according to the manufacturer’s instructions (BD Biosciences, San Diego, CA USA). Briefly, cells were washed twice with PBS and digested with 0.25% trypsin for 5 min. Cell were then centrifuged at 1500 g for 5 min and incubated in 0.5 ml of Binding Buffer (10 mM HEPES pH 7.4, 150 mM NaCl, 2.5 mM CaCl2, 1 mM MgCl2, and 4% BSA), with 4 μg/ml Annexin V-FITC for 15 min. Cells were then washed in PBS and resuspended in Binding Buffer with 0.6 μg/ml Propidium Iodide (PI). Data acquisition and analysis were performed in a FACSCalibur flow cytometry system (BD Biosciences, San Jose, CA, USA) using Cyflogic software V1.2.1 (Perttu Terho, Mika Korkeamaki, CyFlo Ltd, Turku, FINLAND). A total of 5×10^4^ events were collected for each sample.

### Western blot analysis

Cells were lysed in a lysis buffer (50 mM Tris pH 7.4, 0.8 M NaCl, 5 mM MgCl2, 0.1% Triton X-100,) containing Protease Inhibitor and Phosphatase inhibitor Cocktail (Roche, Diagnostics; Mannheim, Germany), incubated on ice for 15 min and cleared by microcentrifugation. Protein concentrations were measured by BioRad™ protein assay kit (Richmond, CA, USA). Cell proteins extracts (20 μg) were separated by sodium dodecyl sulfate-polyacrylamide gel electrophoresis (SDS-PAGE) and then transferred onto a PVDF membrane. Thereafter, nonspecific binding was blocked with 5 % of BSA in TTBS for 1 h at room temperature. Membranes were then incubated overnight at 4 °C with primary antibodies. After washing in TTBS, membranes were incubated with peroxidase-conjugated anti-mouse or anti-rabbit secondary antibodies (1:2000) for 2 h at room temperature. The immune complex was visualized with an ECL system (Cell Signaling Technology).

### siRNA tranfections

Cells were transfected in 1 ml OPTIMEN containing 4 μg lipofectamine 2000 (Invitrogen, Carlsbad, CA), with 100 nM AMPK specific small interfering RNA (siRNA) duplexes siRNA (Ambion-Life Technologies, Carlsbad, CA, USA) or control scrambled RNA for 12 h according to manufacturer’s protocols (Invitrogen, Carlsbad, CA). At 24 h after transfection, the medium was removed and replaced for DMEM containing 10% fetal bovine serum. At dedicated time points after transfection, cells were used for MTT cell viability assays or Western blot.

### Ethics statement

Animal experiments have followed the ARRIVE guidelines and have been carried out in accordance with the U.K. Animals (Scientific Procedures) Act, 1986 and associated guidelines, EU Directive 2010/63/EU for animal experiments. The procedure was approved by Alcalá University Ethical Commission and by the Ethical Commission of the Comunidad de Madrid (procedure PROEX 241/15). All animal studies were conducted in accordance with the Spanish institutional regulation (RD 53/2013) for the housing, care and use of experimental animals and met the European Community directives regulating animal research. Recommendations made by the United Kingdom coordinating Committee on Cancer Research (UKCCCR) have been kept carefully. To assess the welfare of animals a panel of 10 indicators were recorded each day. When adverse effects, pain or distress were appreciated in the animals (score of 15 out of 40) the humane endpoint was applied.

### Animal studies

Athymic nude-Foxn1 (nu/nu) four week-old mice were purchased from Envigo RMS (Barcelona, Spain) and housed in a laminar air-flow cabinet under pathogen-free conditions on a 12-h light/dark schedule at 21-23°C and 40-60% humidity with access to food pellets and tap water ad libitum. 4 animals were housed by cage. G Power analysis was used to calculate sample size [32], according to our previous data and experience and considering two tails effect and a significance level of 5%. Hepatocarcinoma tumors were induced in athymic mice by subcutaneal injection of 5×10^6^ HepG2 or Huh-7 cells. Two weeks after transplantation, tumors had grown to an average volume of 70 mm^3^. Mice were then randomly divided into four experimental groups of 6 animals each, which daily received the following treatments as i.p. injections: Vehicle (DMSO), 2.5 mg/Kg Capsaicin (CAP), 30 mg/Kg sorafenib (SF) or 2.5 mg/Kg Capsaicin + 30 mg/Kg sorafenib (CAP + SF). Tumor sizes were measured every day and calculated using the formula V(mm^3^) = 1/2(Length × Width^2^). At the end of the study, the mice were sacrificed by placing them in a CO2 gas-filled chamber, and the excised tumors were recovered and weighted.

### Combined drug analysis

Drug interaction was determined using the combination index (CI)-isobologram equation that allows quantitative determination of drug interactions, where CI < 1 implied synergism, CI=1 additive, and CI >1 implied antagonism [16, 17]. Compusyn^©^ version 1.0 software (ComboSyn, Inc. Paramus, NJ, USA) was used to generate the dose-response curves, dose-effect analysis, and CI-effect plot.

### Statistical analysis

The GraphPad Prism 6 software (GraphPad software Inc., la Jolla, CA, USA) was used to calculate statistical significance. Interaction significance of two treatments were determined at fixed concentration of each treatment using two-way analysis of variance (ANOVA) and multiple comparisons between two groups were performed using Dunett, Tukey or Sidak tests. The results were reported as mean ± S.E.M. or S.D. as indicated in figure caption, of at least three independent experiments and, the results for parameters with a significance level of P < 0.05 were considered as significant.

## SUPPLEMENTARY MATERIALS FIGURES AND TABLES


