# Supplementary material for: Capsaicin exerts synergistic antitumor effect with sorafenib in hepatocellular carcinoma cells through AMPK activation
Source: Oncotarget. 2017 Sep 23;8(50):87684–98. doi: 10.18632/oncotarget.21196 (PMC5675664; doi:10.18632/oncotarget.21196)
Supplement: Supplementary file 1 [file oncotarget-08-87684-s001.pdf]

Capsaicin exerts synergistic antitumor effect with Sorafenib in Hepatocellular carcinoma cells through AMPK activation

SUPPLEMENTARY MATERIALS

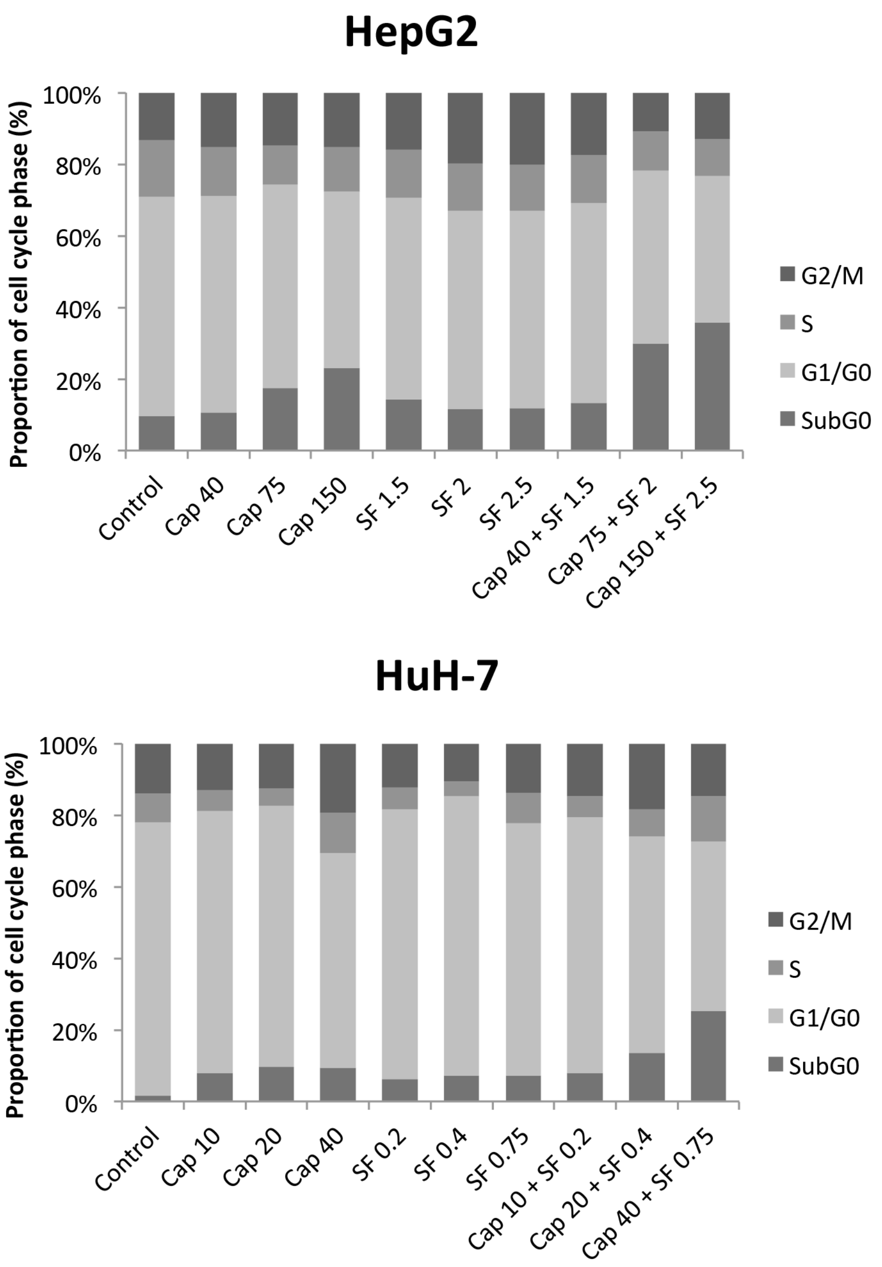

Supplementary Figure 1: Cell cycle progression in HepG2 and HuH-7 cell lines treated with different doses of capsaicin, sorafenib or a combination. Cells were exposed to indicated concentrations of compounds for 24 h. Flow cytometric analysis after propidium iodide (PI) staining was applied to determine the alterations in cell cycle distributions.Columns in the diagram depict cell cycle phase distribution in HCC cells.
